# Cannabis Use as Risk or Protection for Type 2 Diabetes: A Longitudinal Study of 18 000 Swedish Men and Women

**DOI:** 10.1155/2016/6278709

**Published:** 2016-10-24

**Authors:** A. K. Danielsson, A. Lundin, A. Yaregal, C. G. Östenson, P. Allebeck, E. E. Agardh

**Affiliations:** ^1^Department of Public Health Sciences, Karolinska Institutet, Stockholm, Sweden; ^2^Department of Molecular Medicine and Surgery, Karolinska Institutet, Stockholm, Sweden; ^3^Centre for Epidemiology and Community Medicine, Stockholm County Council, Stockholm County, Sweden

## Abstract

*Aims*. Whether or not cannabis use may increase or decrease the risk of type 2 diabetes is not clear. We analyzed the association between cannabis and subsequent type 2 diabetes and if a potential positive or reverse association persisted after controlling for potential confounders.* Methods*. In this population-based cohort study, 17,967 Swedish men and women (aged 18–84 years), who answered an extensive questionnaire in 2002 (including questions on cannabis use), were followed up for new cases of type 2 diabetes (*n* = 608) by questionnaire (in 2010) and in health registers during 2003–2011. Odds ratios (ORs) with 95% CIs were estimated in a multiple logistic regression analysis. Potential confounders included age, sex, BMI, physical inactivity, smoking, alcohol use, and occupational position.* Results*. The crude association showed that cannabis users had a reduced risk of type 2 diabetes OR = 0.68 (95% CIs: 0.47–0.99). However, this inverse association attenuated to OR = 0.94 (95% CIs: 0.63–1.39) after adjusting for age.* Conclusions*. The present study suggests that there is no association between cannabis use and subsequent type 2 diabetes after controlling for age. To make more robust conclusions prospective studies, with longer periods of follow-up and more detailed information about cannabis use, are needed.

## 1. Introduction

Globally, cannabis is among the most widely used illicit drugs with an estimated 180 million people aged 15–64 years using it for nonmedical purposes [1]. In recent decades, the use of cannabis has been growing much more rapidly than the use of other substances, and the most rapid growth since 1960 has occurred in high-income countries [1]. The current wave of decriminalization in, for example, the United States may lead to more widespread use, and it is important to address the potential cannabis-associated adverse health effects that may begin to occur in the population at a greater frequency [2]. Previous studies have linked cannabis use to a variety of psychiatric and somatic disease including anxiety [3], schizophrenia [4, 5], depression [6], dependence [7], chronic bronchitis [8], respiratory infections [9], lung cancer [10], myocardial infarction [2], and stroke [11].

Whether or not cannabis use may increase or decrease the risk of developing type 2 diabetes is still pending. The question is rarely studied and results are conflicting. The most well-established risk factors for type 2 diabetes are overweight and obesity, physical inactivity, unhealthy food intake, and increasing age [12]. Other important risk factors include smoking, high alcohol intake, genetics, and a family history of diabetes [12–14]. Thus, while the association between several life-style factors and type 2 diabetes is established, possible associations between cannabis use and type-2 diabetes remain to be clarified.

The two main ingredients in the cannabis plant are cannabidiol (CBD) and tetrahydrocannabinol (THC), with seemingly different characteristics. Both CBD and THC belong to a class of compounds known as cannabinoids. Cannabinoids are chemical components that act on cannabinoid receptors in cells that modulate neurotransmitter release in the brain [15]. Animal studies have shown that cannabinoids, primarily THC, stimulate appetite through activation of certain CB_1_ receptors [16] and may play a role in compulsive eating behavior [17], while others have reported a significant reduction, or delayed onset, in the development of type 2 diabetes in CBD treated mice [18, 19]. There is, however, little research evidence to support this direct effect of cannabis on humans.

Activation of CB_1_ receptors has been shown to promote food craving and hyperphagia also in humans [20]. This led to the development of endocannabinoid receptor antagonists as therapy for obesity and type 2 diabetes [21]. However, these drugs were withdrawn early from the market due to severe psychiatric adverse effects.

One cross-sectional study from the US showed that previous, current light and heavy cannabis users had relatively lower odds of diabetes compared with nonusers [22]. It has been suggested that cannabis use may lower the risk of diabetes by lowering fasting insulin levels, as well as the risk of insulin resistance [22, 23]. Despite the fact that the use of cannabis often is associated with a unfavorable life-style, such as higher intake of calories, a higher tobacco smoking rate, higher consumption of alcohol, and illicit drugs [24], studies have also shown that cannabis use may lower body mass index (BMI) [25] and reduce the prevalence of obesity [26]. Moreover, a recent meta-analysis including eight separate US samples reported that active cannabis use was inversely associated with type 2 diabetes [27]. However, the included samples were all from cross-sectional studies and, as stressed by the authors, more research is needed [27].

In contrast, a recent longitudinal study from the US that assessed cannabis use in young adulthood and the risk of prediabetes and type 2 diabetes in middle adulthood found that lifetime cannabis use of 100 times or more was associated with higher odds for prediabetes compared with never use [28]. This study did not, however, report a significant association between cannabis use and later development of type 2 diabetes.

Thus, previous studies are scarce, all conducted in the US, many comprising the same study population, and mostly conducted with a cross-sectional design. Furthermore, results are inconclusive. In this study, we will therefore make use of a population-based cohort of Swedish men and women to examine the possible association between cannabis use and type 2 diabetes. More specifically, we aim to find out (1) whether cannabis use increases or decreases the risk of type 2 diabetes and (2) whether a possible association persists after controlling for potential confounding variables, such as age, sex, BMI, tobacco smoking, alcohol consumption, occupational position, and physical inactivity.

## 2. Materials and Methods

### 2.1. Study Population

The population included in our study, that is, the Stockholm Public Health Cohort (SPHC), has been described in detail elsewhere [29]. In brief, we used data from the cohort with a baseline survey that took place in 2002 comprising 31,182 (equal to a response rate of 63%) randomly selected Swedish men and women aged 18 to 84 years living in Stockholm County. The participants were followed up and reinvestigated in 2010 (*n* = 19,327; 62% retention rate).

Those without information on cannabis use at baseline (*n* = 133) and those who reported diabetes in the SPHC questionnaire at baseline, or with a confirmed diagnosis in the medical registers in 2002 or ten years before, were excluded for further analyses (*n* = 1361). The final study sample (*n* = 17,833) included 7533 men and 10,300 women.

### 2.2. Data Collection

At baseline in 2002, participants answered a detailed questionnaire covering somatic and psychological health, demographics, family situation, housing, work environment, socioeconomic position (SEP), and lifestyle factors (e.g., alcohol use, tobacco smoking, physical activity, height and weight, and cannabis use).

The participants of the cohort were via their personal identification numbers linked to the national patient register that records all admissions to hospitals in Sweden, including both in- and outpatients (PR) and to the regional primary healthcare register (VAL). PR is estimated to have a high coverage from 1987 and onwards with respect to most diseases and the VAL database is a great complement to the PR since many diabetics are diagnosed and treated in the primary healthcare in Sweden.

### 2.3. Measures

#### 2.3.1. Exposure: Cannabis Use

In 2002 the participants were asked if they “had ever smoked hashish.” The response alternatives were “no,” “yes, more than one year ago,” “yes, during the past year,” and “yes during the past month.” In the analyses we categorized these into never (no) and ever users (e.g., yes, more than one year ago; yes, during the past year; and yes during the past month), due to limited number of cases reporting hashish during the past month and past year. Hashish will herein be referred to as cannabis.

#### 2.3.2. Outcome: Type 2 Diabetes

The outcome variable, type 2 diabetes was obtained during follow-up by self-reported (SPHC 2010) as well as diagnoses recorded in the healthcare databases (2003–2011). The self-reported data was obtained by survey responses to the question “have you ever been diagnosed with diabetes by a medical doctor?” (“yes” or “no”). The registered based data was based on the identification of persons diagnosed with type 2 diabetes by the ICD codes E11–E13.

#### 2.3.3. Potential Confounders

The choice of potential confounders was based on previous studies on cannabis and type 2 diabetes and availability in the questionnaire. These included age, sex, BMI, tobacco smoking, alcohol consumption, occupational position, and physical activity.

*Age* was categorized into the following categories (18–44, 45–64, and 65–84).* BMI* was based on self-reported weight and height and calculated as weight in kilograms divided by the square of height in meters and categorized into three groups (≤24.9, 25–29.9, and ≥30), according to WHO standards.* Tobacco smoking* was based on the question “do you smoke on a daily basis?” and refers to all sorts of smoking, such as cigarettes, cigarillos, cigars, and pipes, with two responding alternatives, “yes” or “no.”* Alcohol consumption* was measured with questions on the total quantity of beer, wine, and spirits consumed during an average week the past year. We estimated the amount of 100% alcohol in grams per week and transformed the volume into standard drinks, where one drink contains 12 grams of alcohol. We created 5 cut-off groups from the percentile distribution among all respondents: no alcohol consumption; >0 to <3.5 drinks per week; ≥3.5 to <7 drinks per week; ≥7 to <12 drinks per week; and ≥12 drinks per week.* Occupational position* was based on self-reported current or previous occupational titles and classified as low (“unskilled and skilled workers”), middle (“low-level nonmanual employees”), and high (“high and medium level nonmanual employees and self-employed”) according to Statistics Sweden's socioeconomic position classification scheme. Some persons who did not report any occupation (students, retried, housewives, unemployed, or disability pensionaries) were excluded from further analysis.* Physical activity* was based on the question “how much have you exercised and exerted yourself physically in the past 12 months?” with 4 response options including low exercise activity, moderate exercise, moderate regular exercise, and regular exercise and training. We categorized those who responded to have low exercise as physically inactive and the rest as physically active.

### 2.4. Statistical Analyses

To examine the possible association between cannabis use and type 2 diabetes, odds ratios (ORs) with 95% Confidence Intervals (CIs) were estimated in a logistic regression model. First, we assessed the crude estimate. Second, the effect of each potential confounding factor, measured at baseline, was checked by controlling for them one at a time in the association between cannabis use and type 2 diabetes. Finally, we included all confounders in the same model. A variable was considered a confounder and included in the final model, if changing the OR with more than 10% [30]. In all estimated ORs we used a system of weights created for the SPHC to account for nonresponse. These weights are constructed on the basis of available auxiliary variables from different national registers and their covariation with survey data, such as age, sex, country of birth, marital status, income, education, sick-leave benefits, and stratum. Consequently, by including these weights the overrepresentation of, for example, well-educated women in the material was taken into account. All statistical analyses were employed using SAS (version 9.3) software package, and the logistic regression was conducted using the SURVEY LOGISTIC procedure.

## 3. Results

The baseline characteristics of the study participants are presented in absolute, nonweighted numbers and proportions (Table 1). Out of 17,833 participants, 14.3% had ever used cannabis. Among these, 1317 (51.8%) were men and 1227 (48.2%) were women. Nearly 64% of the cannabis users were within the age range of 18–44 years, compared to approximately 40% of never users.

Cannabis users were more likely to smoke tobacco (23.5%) compared to never users (13.1%) and to have a high consumption of alcohol (41.3%) compared to never users (22.1%). Those with more recent use of cannabis (past month or past year) were also more likely to smoke tobacco and have a high consumption of alcohol compared to those who used cannabis more than one year ago. In terms of BMI status, 66.1% of the cannabis users had a BMI of ≤24.9 compared to 58.5% of never users. Moreover, approximately 77% of those who smoked cannabis the past month or past year had a BMI of ≤24.9, compared to 65% that used cannabis more than one year ago, implying that recent cannabis users were leaner. The distribution of physical inactivity and occupational position was similar between ever users and never users, while past month users were more likely to be physically inactive and have a low occupational position compared to never users.

Having ever used cannabis was inversely associated with type 2 diabetes, OR = 0.68 (95% CIs: 0.47–0.99) (Table 2). This inverse association persisted after adjusting for sex OR = 0.65 (95% CIs: 0.44–0.95) but was attenuated to OR = 0.94 (95% CIs: 0.63–1.39) after adjusting for age. When we adjusted for all confounders that changed the OR more than 10% (age, tobacco smoking, BMI, and occupational position) in the same model, the OR was 0.94 (95% CIs: 0.63–1.42) and hence virtually the same as when adjusting for age alone.

## 4. Discussion

Our study suggests that there is no significant association between cannabis use and subsequent type 2 diabetes after controlling for age.

Given the scarcity in previous studies, these findings are important. While cross-sectional studies mainly show inverse association between cannabis use and type 2 diabetes [27], our results are in line with Bancks et al.'s longitudinal study [28]. Findings from cross-sectional studies may be due to reverse causation: that is, persons with diabetes may have lower likelihood to use cannabis due to health awareness. It is puzzling, however, that cannabis use in the above-mentioned longitudinal study was associated with an increased risk of prediabetes [28]. Type 2 diabetes is insidious and develops gradually through prediabetes under the influence of genetics, aging, and adverse lifestyle factors [12], and one of Bancks et al.'s hypotheses was that cannabis may have a greater impact on glucose metabolism when traditional risk factors do not dominate over the effect of cannabis [28]. Unfortunately, we had no information on prediabetes and could therefore not investigate if the same would apply in this Swedish population.

As in previous research, cannabis users in our study, at least those smoking cannabis during the past month, were more likely to have unhealthy behaviors, such as tobacco smoking, high consumption of alcohol, and physical inactivity [24]. In contrast, cannabis users had lower BMI levels compared to nonusers. Studies on the association between cannabis use and BMI are conflicting, showing inverse [26], positive [31], and no associations [32]. Accordingly, the impact of BMI on the association between cannabis and type 2 diabetes is uncertain, and whether BMI acts as a mediator or confounder needs to be further studied. Although cannabis users in our study had lower levels of BMI at baseline, cannabis was not significantly related to any weight change between the years 2002 and 2010 (sex and age-adjusted weight change coefficient in cannabis users from linear regression = −0.26, SE = 0.26, *p* value = 0.31). Regulation of the BMI is closely related to the whole body energy balance, and it is now accepted that cannabinoid receptor agonists not only facilitate energy intake but also can enhance energy storage into the adipose tissue and reduce energy expenditure by influencing both lipid and glucose metabolism [21]. In this way, cannabis can impact not only BMI, but also insulin sensitivity differently in different tissues like adipose tissue and the liver [33]. Taken together, these rather complex interrelationships may partly explain the results of previous studies, showing varying effects by the compound on BMI and risk of type 2 diabetes.

It should be noted that we had no detailed information about duration or frequency of cannabis use and the way we estimated ever users by combining past month, past year, and more than one year ago is simplistic. We did, although based on very few cases, check the dose-response association and found that the age-adjusted ORs of type 2 diabetes were 0.97 if having used cannabis more than one year ago and decreased to 0.74 when combining cannabis use during the past month and past year compared to never users. These associations were nonsignificant. Cannabis use in Stockholm and Sweden is not as prevalent as in other countries, for example, the US or the UK, and it maybe that our sample is too small to reach significance. Thus, although the prevalence of cannabis use in our cohort is similar to national estimates [34], the possibility of a reverse association between cannabis use and subsequent type 2 diabetes cannot be ruled out.

There are some methodological issues that should be acknowledged. First, our dichotomization of the information on cannabis use may have resulted in a dilution of the possible effects from cannabis. In addition to this, self-reported cannabis use may be subject to underreporting. Other health surveys have shown that nonrespondents have an increased risk of alcohol-, drug-, and smoking-related mortality and morbidity compared with respondents [35]. It is therefore possible that we may have underestimated the proportion of people who use cannabis. Furthermore, one of the answers to the cannabis question was about use over one year ago, which could imply recall bias. However, since all subjects were diabetes-free at baseline, possible misclassification should not lead to an overestimation of the ORs.

Strengths of our study were that all participants were healthy at baseline and new diabetes cases were identified and ascertained by questionnaire and by health registers at follow-up. Only 60 persons (approximately 1 percent) that reported diabetes in the questionnaire at follow-up did not appear in the health registers. The reason for this is not known, but it is possible that some cases are prediabetics, who instead received advice from a medical doctor about lifestyle changes. Another strength was the extensive survey data on possible confounding factors, and the fact that the inverse association disappeared after controlling for them confirmed their importance.

## 5. Conclusions

To conclude, our results indicate no association between cannabis use and subsequent type 2 diabetes. To make more robust conclusions, longitudinal studies with longer follow-up and more detailed information about cannabis use are needed. Our findings are important to confirm, since negative effects of cannabis have been reported in relation to a number of adverse health outcomes.

## Figures and Tables

**Table 1:** Baseline characteristics of participants in 2002 by cannabis use: The Stockholm Public Health Cohort (2002–2010).

	All	Cannabis use				
		Past month	Past year	>1 year ago	Ever^†^	Never
Total	17,833 (100)	65 (0.4)	184 (1.0)	2,295 (12.9)	2,544 (14.3)	15,289 (85.7)
Sex						
Men	7,533 (42.2)	39 (60)	95 (51.6)	1,183 (51.5)	1,317 (51.8)	6,216 (40.7)
Women	10,300 (57.8)	26 (40)	89 (48.4)	1,112 (48.5)	1,227 (48.2)	9,073 (59.3)
Type 2 diabetes^*∗*^						
Yes	604 (3.4)	2 (3.1)	3 (1.6)	55 (2.4)	60 (2.4)	544 (3.6)
No	17,229 (96.6)	63 (96.9)	181 (98.4)	2,240 (97.6)	2,484 (97.6)	14,745 (96.4)
Age-groups (years)						
18–44	7,713 (43.3)	53 (81.6)	157 (85.3)	1,414 (61.6)	1,624 (63.8)	6,089 (39.8)
45–64	7,532 (42.2)	11 (16.9)	27 (14.7)	867 (37.8)	905 (35.6)	6,627 (43.4)
65–84	2,588 (14.5)	1 (1.5)	0 (0)	14 (0.61)	15 (0.6)	2,573 (16.8)
BMI						
≤24.9	10,629 (59.6)	50 (76.9)	142 (77.2)	1,490 (64.9)	1,682 (66.1)	8,947 (58.5)
25–29.9	5,811 (32.6)	14 (21.5)	34 (18.5)	646 (28.2)	694 (27.3)	5,118 (33.5)
≥30	1,393 (7.8)	1 (1.54)	8 (4.3)	159 (6.9)	168 (6.6)	1,225 (8.0)
Tobacco smoking						
No	15,161 (85.4)	30 (46.2)	116 (63.4)	1,789 (78.4)	1,935 (76.5)	13,226 (86.9)
Yes	2,583 (14.6)	35 (53.9)	67 (36.6)	494 (21.6)	596 (23.5)	1,987 (13.1)
Alcohol use (drinks/week)						
0	968 (6.0)	5 (7.9)	4 (2.3)	73 (3.4)	82 (3.4)	886 (6.5)
>0–<3.5	3,341 (20.7)	4 (6.4)	5 (2.9)	305 (14)	314 (13.0)	3,027 (22.0)
≥3.5–<7	3,765 (23.3)	8 (12.7)	12 (6.9)	417 (19.1)	437 (18.0)	3,328 (24.2)
≥7–<12	4,040 (25.0)	8 (12.7)	36 (20.8)	543 (24.9)	587 (24.3)	3,453 (25.4)
≥12	4,039 (25.0)	38 (60.3)	116 (67.1)	844 (38.7)	998 (41.3)	3,041 (22.1)
Occupational position						
High	9,201 (54.0)	19 (31.7)	64 (40.5)	1253 (57)	1,336 (55.3)	7,865 (53.9)
Middle	2,788 (16.4)	7 (11.7)	30 (19.0)	301 (13.7)	338 (14.0)	2,450 (16.8)
Low	5,030 (29.6)	34 (56.7)	64 (40.5)	645 (29.3)	743 (30.7)	4,287 (29.3)
Physical activity						
Active	15,225 (86.9)	47 (72.3)	157 (86.7)	1,927 (84.7)	2,131 (84.6)	13,094 (87.3)
Inactive	2,299 (13.1)	18 (27.7)	24 (13.3)	347 (15.3)	389 (15.4)	1,910 (12.7)

Data are presented as nonweighted numbers (*n*) and percent (%).

^†^Ever users (combined past month, past year, and >1 year ago).

Missing data for 133 persons with regard to cannabis use.

^*∗*^Type 2 diabetes cases (2003–2011). Four cases of type 2 diabetes were missing due to missing data on cannabis use.

**Table 2:** Odds ratios (ORs) and 95% Confidence Intervals (CIs) for the association between cannabis use in 2002 and subsequent type 2 diabetes (2003–2011).

	Cannabis use		
	Never	Ever	
		ORs	(95% CIs)
*Crude estimate*	1	0.68	(0.47–0.99)
*Adjusted for*			
Sex	1	0.65	(0.44–0.95)
Age	1	0.94	(0.63–1.39)
Tobacco smoking	1	0.60	(0.41–0.87)
Physical inactivity	1	0.66	(0.46–0.97)
BMI	1	0.75	(0.51–1.10)
Alcohol use	1	0.67	(0.44–1.02)
Occupational position	1	0.75	(0.51–1.09)
Multiadjusted^*∗*^	1	0.94	(0.63–1.42)

^*∗*^Adjusted for age, BMI, tobacco smoking, and occupational position, which changed the OR > 10%.
